# Supplementary material for: Projected losses of global mammal and bird ecological strategies
Source: Nat Commun. 2019 May 23;10:2279. doi: 10.1038/s41467-019-10284-z (PMC6533255; doi:10.1038/s41467-019-10284-z)
Supplement: Supplementary file 1 — Supplementary Information [file 41467_2019_10284_MOESM1_ESM.pdf]

Supplementary Information

Projected losses of global mammal and bird ecological strategies

Cooke et al.

Supplementary Methods 3

Supplementary Figure 1: Ecological strategy surfaces for mammals and birds for each pairwise combination of the five principal components 5

Supplementary Figure 2: Ecological strategy surfaces for mammals and birds for each of the 25 imputed datasets 6

Supplementary Table 1: Mean variable loadings (across 25 imputed datasets) resulting from the principal components analysis 7

Supplementary Figure 3: The ecological strategy surface for mammals and birds with species locations, scientific names and image sources 8

Supplementary Figure 4: Principal coordinates analysis (PCoA) of diet categories for mammals and birds 9

Supplementary Figure 5: The ecological strategy surface for mammals and birds with categorical divisions 10

Supplementary Figure 6: Pairwise correlations between five traits for mammals and birds 11

Supplementary Table 2: Percentage occupation by the observed ecological strategy space of the mean of 999 null strategy spaces generated from the assumptions of each null model, for each taxonomic group 12

Supplementary Table 3: Contributions of different taxonomic and morphological groups to ecological strategy space 13

Supplementary Figure 7: Plots showing the results of the permutation tests 14

Supplementary Figure 8: The ecological strategy surface for mammals and birds under the data deletion approach 15

Supplementary Figure 9: The ecological strategy space for mammals and birds under 100-year extinction scenarios under the data deletion approach 16

Supplementary Figure 10: Plots showing the results of the permutation tests under the data deletion approach 17

Supplementary Figure 11: The ecological strategy surface for mammals and birds when including two synthetic diet traits 18

Supplementary Figure 12: The ecological strategy space for mammals and birds under 100-year extinction scenarios when excluding DD species 19

Supplementary Figure 13: Plots showing the results of the permutation tests when excluding DD species 20

Supplementary Figure 14: The ecological strategy space for mammals and birds under 100-year extinction scenarios with predicted extinction probability for DD species 21

Supplementary Figure 15: Plots showing the results of the permutation tests when predicting extinction probability for DD species 22

Supplementary Figure 16: Ecological strategy surface for mammals 23

Supplementary Figure 17: Ecological strategy surface for birds 24

Supplementary references 24

Supplementary Methods

Traits

We extracted three categorical traits for post-analysis interpretations[1]: flight capability (volant/non-volant), diel activity (diurnal/nocturnal) and diet guild (an aggregated diet score) (Supplementary Figure 5). For diet guild, species were classified into five groups according to their primary diet[2]: plant/seed, fruit/nectar, invertebrates, vertebrates (including carrion), and omnivore (score of ≤ 50 in the four other diet categories).

Ecological strategy surface

We used only the first principal component from the diet PCoA for our main analyses, so that each trait dimension had equal weight. However, for comparison we provide the ecological strategy surface when including two synthetic diet traits (first and second principal components from diet PCoA; Supplementary Figure 4) (Supplementary Figure 11), which is very similar to when we only include one synthetic diet trait (main text Fig. 1). Moreover, diet guild showed clear patterning across the ecological strategy surface (Supplementary Figure 5d), indicating that the use of a single diet axis sufficiently captured the variation in species diets. For instance, carnivores show distinct separation on the ecological strategy surface (Supplementary Figure 5d, see marginal plot on PC1), despite the low importance of carnivory in the PCoA (Supplementary Figure 4). We also generated ecological strategy surfaces for mammals (Supplementary Figure 16) and birds (Supplementary Figure 17) separately.

Overall our results and conclusions for the ecological strategy surface were similar (i) with and without imputed data (compare main text Fig. 1 and Supplementary Figure 8; Supplementary Figure 2), and (ii) with one or two synthetic diet traits (compare main text Fig. 1 and Supplementary Figure 11).

Extinction scenarios

For simplicity, in the main extinction analyses we treated DD species as LC[3,4]. However, in reality, an unknown proportion of DD species are not at risk of extinction whilst others are likely to be threatened. To evaluate the impact of the extinction probability for DD species we implemented two alternative scenarios: excluding DD species (Supplementary Figures

12, 13) and assigning an average predicted extinction probability to DD species (Supplementary Figures 14, 15), based on previous research[5]. To calculate an extinction probability for DD species we first calculated the average extinction probability of threatened species at the same ratio of CR:EN:VU (380:863:1254 species) as for the set of species for which threat categories are known[6]. Thus, the average extinction probability for threatened species was 0.433 = ((380 CR species * 0.999) + (863 EN species * 0.667) + (1254 VU species * 0.1)) / (380 + 863 + 1254). We then did the same for non-threatened species ((1300 NT species * 0.01) + (10963 LC species * 0.0001)) / (1300 + 10963) = 0.001. 64% of DD mammal species were previously predicted to be threatened, using machine learning tools[5]. As the majority of our DD species were mammals (665 DD mammal species, 59 DD bird species) we applied this value across our 724 DD species. So we multiplied the average extinction probability for threatened species (0.433) by the proportion of DD species predicted to be threatened (0.64)[5] and multiplied the average extinction probability for non-threatened species (0.001) by the proportion of DD species predicted to be non-threatened (0.36), and then summed the total extinction probability, resulting in an extinction probability of 0.277 for DD species. Thus, DD species were assigned an average predicted extinction probability that falls between VU and EN.

The IUCN Red List is, of course, not a perfect predictor of the future state of the biosphere, but it represents our best and most comprehensive assessment of the probability that any given species will go extinct in the near future[7]. Here we consider extinction only (disregarding possible speciation), because our focus is on the impact of current high rates of extinction over relatively short time frames (100 years), for which little speciation may be expected.

Overall our results and conclusions for the extinction analyses were similar (i) with and without imputed data (compare main text Fig. 3 and Supplementary Figure 9; plus, compare Supplementary Figure 7 and Supplementary Figure 10), (ii) with and without DD species (compare main text Fig. 3 and Supplementary Figure 12; plus, compare Supplementary Figure 7 and Supplementary Figure 13), and (iii) when assigning DD species an extinction probability of 0.0001 or 0.277 (compare main text Fig. 3 and Supplementary Figure 14; plus, compare Supplementary Figure 5 and Supplementary Figure 15).

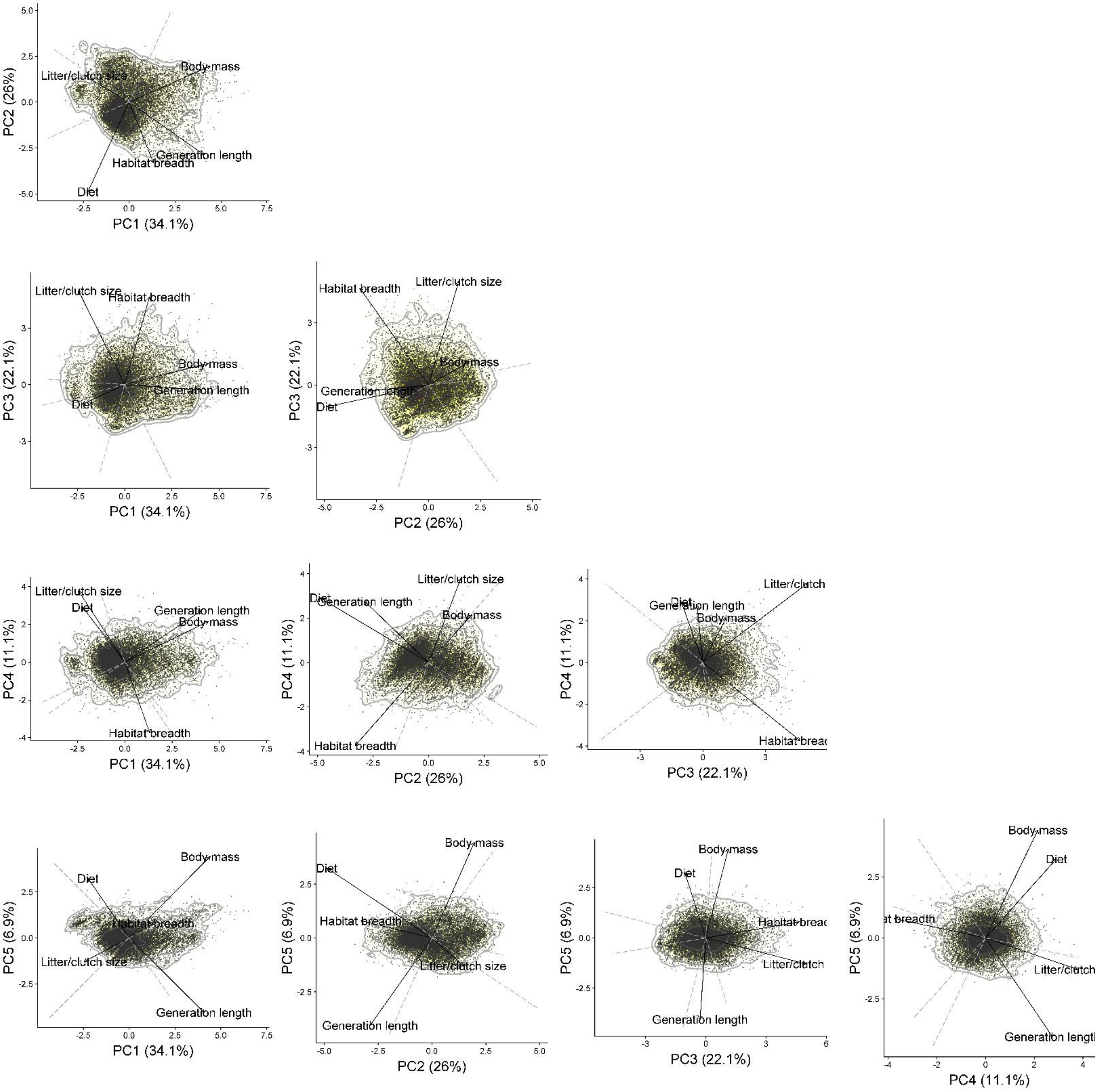

Supplementary Figure 1: Ecological strategy surfaces for mammals and birds (15,484 species) for each pairwise combination of the five principal components. Percentage values represent proportion of the total variation explained by each PC. Source data are provided as a Source Data file.

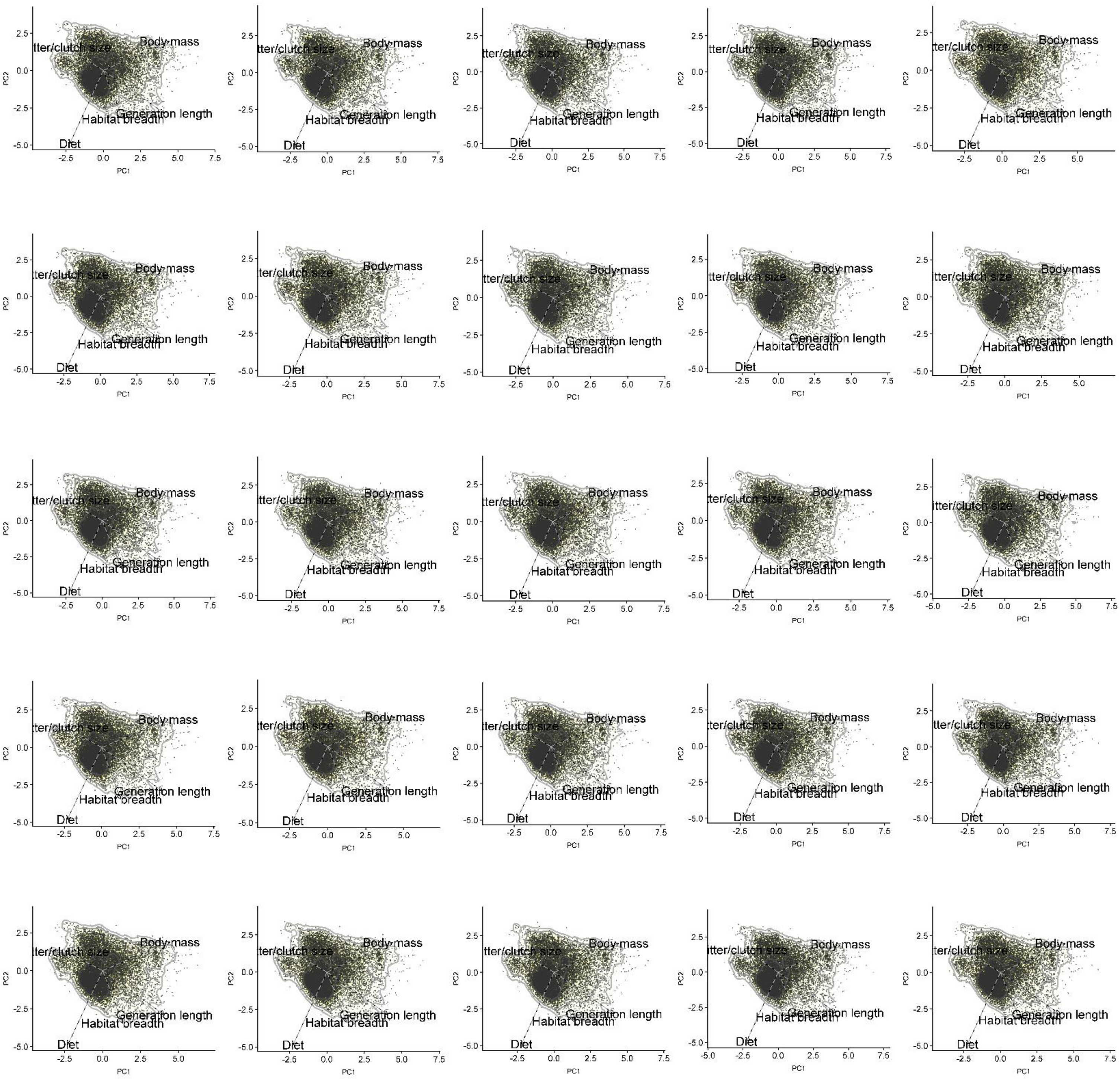

Supplementary Figure 2: Ecological strategy surfaces for mammals and birds (15,484 species) for each of the 25 imputed datasets (Supplementary Methods). The plots show very high similarity across the imputed datasets. Source data are provided as a Source Data file.

Supplementary Table 1: Mean variable loadings (across 25 imputed datasets) resulting from the principal components analysis (PCA; main text Fig. 1). Positive loadings are shown in increasingly dark shades of blue with increasing magnitude, and negative loadings in increasingly dark orange. The proportion of variance of a given trait accounted for can be obtained by squaring the loading.

Trait	PC1	PC2	PC3	PC4	PC5
Body mass	0.63	0.28	0.16	0.31	0.63
Diet	-0.32	-0.70	-0.15	0.42	0.46
Generation length	0.58	-0.41	-0.04	0.39	-0.58
Habitat breadth	0.19	-0.47	0.66	-0.54	0.11
Litter/clutch size	-0.35	0.21	0.71	0.54	-0.18

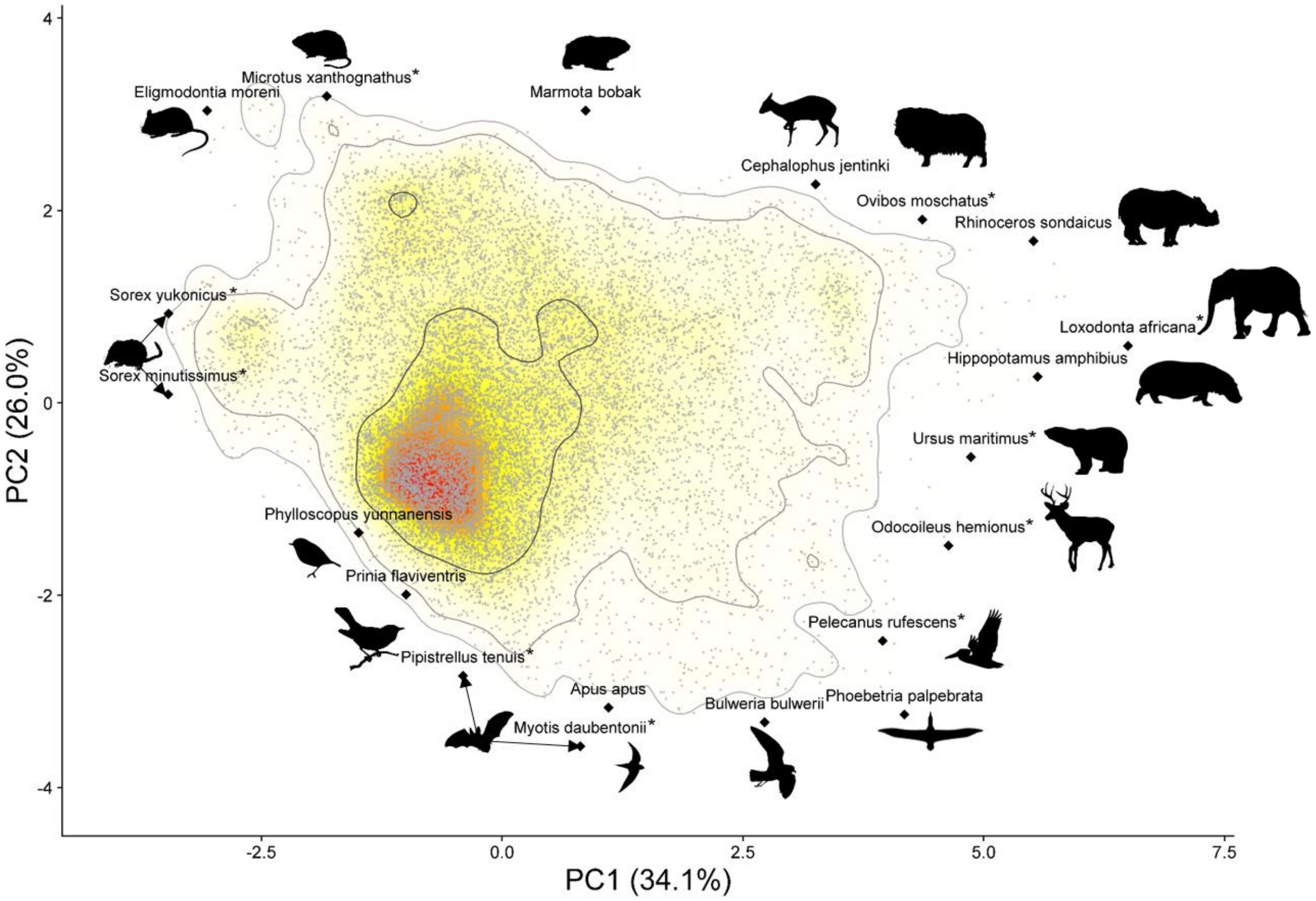

Supplementary Figure 3: The ecological strategy surface for mammals and birds (15,484 species) with species locations, scientific names and image sources. Projection of extant terrestrial mammal and bird species (grey dots) on the surface defined by principal components (PC) 1 and 2 (mean values across 25 imputed datasets; Supplementary Methods). Silhouettes show a selection of species characterising the edges of trait space, with their locations represented by black diamonds and scientific names labelled. Silhouettes with scientific names followed by an * were freely downloaded from PhyloPic (www.phylopic.org), under CC0 1.0 Public Domain Dedication. The rest of the silhouettes were created by the authors in Inkscape. The silhouette of a bat is used to represent the location of two ecological outliers: *Pipistrellus tenuis* and *Myotis daubentonii* and the silhouette of the shrew for *Sorex minutissimus* and *Sorex yukonicus*. Source data are provided as a Source Data file.

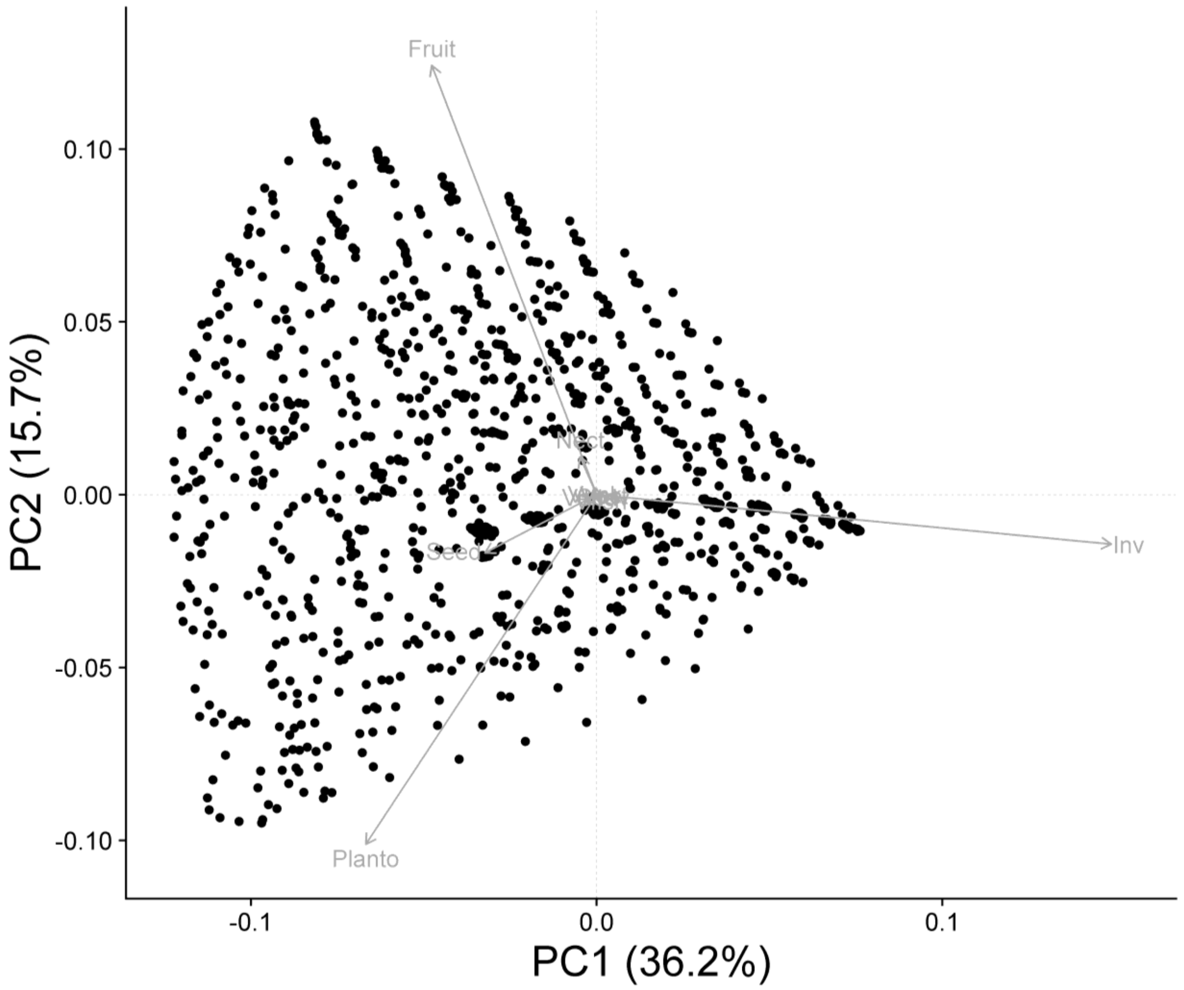

Supplementary Figure 4: Principal coordinates analysis (PCoA) of diet categories for mammals and birds (15,484 species). Arrows indicate direction and weighting of vectors representing the ten diet categories: Inv (invertebrates); Planto (other plant material); Fruit (fruit and drupes); Seed (seed, nuts); Nect (nectar, pollen, gum); Vend (vertebrate endotherms); Vect (vertebrate ectotherms); Vfish (fish); Vunk (vertebrate unknown); Scav (scavenge) - for full descriptions see the EltonTraits 1.0 metadata[2]. Percentage values represent proportion of the total variation explained by each PC. Source data are provided as a Source Data file.

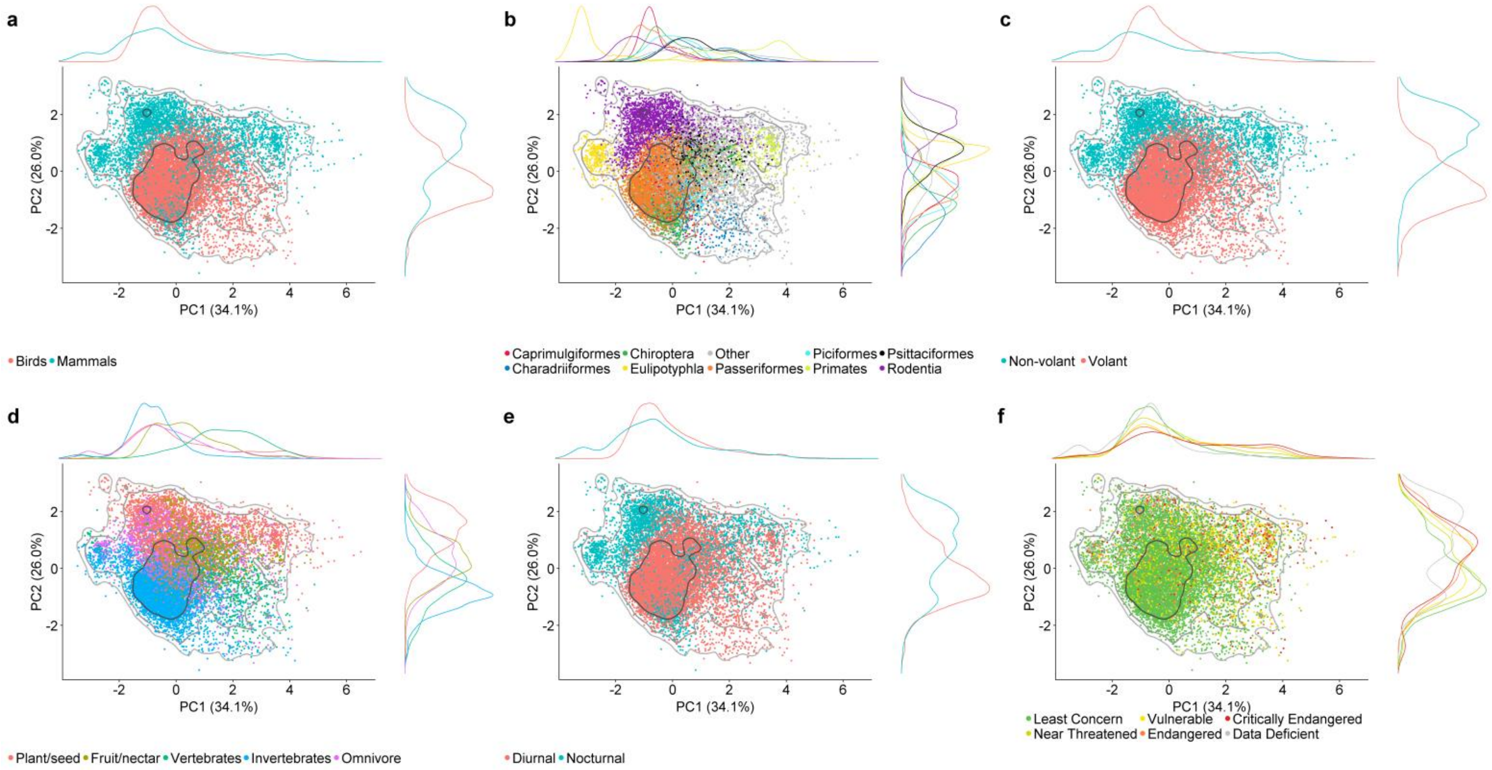

Supplementary Figure 5: The ecological strategy surface for mammals and birds (15,484 species) with categorical divisions. Differentiating categories include (a) Class, (b) the most speciose Orders, (c) flight capability, (d) dietary guild, (e) diel activity and (f) IUCN Red List category. Contours indicate 0.5 (hotspots), 0.95 and 0.99 quantiles of occurrence probability of species across the ecological strategy surface. Marginal plots indicate density distributions of the categories along the principal component axes. The most speciose Orders: Passeriformes - perching birds (5,903 species), Rodentia - rodents (2,206 species), Chiroptera - bats (1,104 species), Caprimulgiformes - nightjars, swifts, hummingbirds, and relatives (586 species), Piciformes - woodpeckers, toucans, puffbirds, and relatives (482 species), Eulipotyphyla - shrews, moles, hedgehogs and solenodons (441 species), Primates - apes, monkeys, lemurs, and relatives (414 species), Psittaciformes - parrots (397 species), and Charadriiformes - waders, gulls, auks, and relatives (374 species); the ‘Other’ category is a group comprised of 52 less speciose Orders. Source data are provided as a Source Data file.

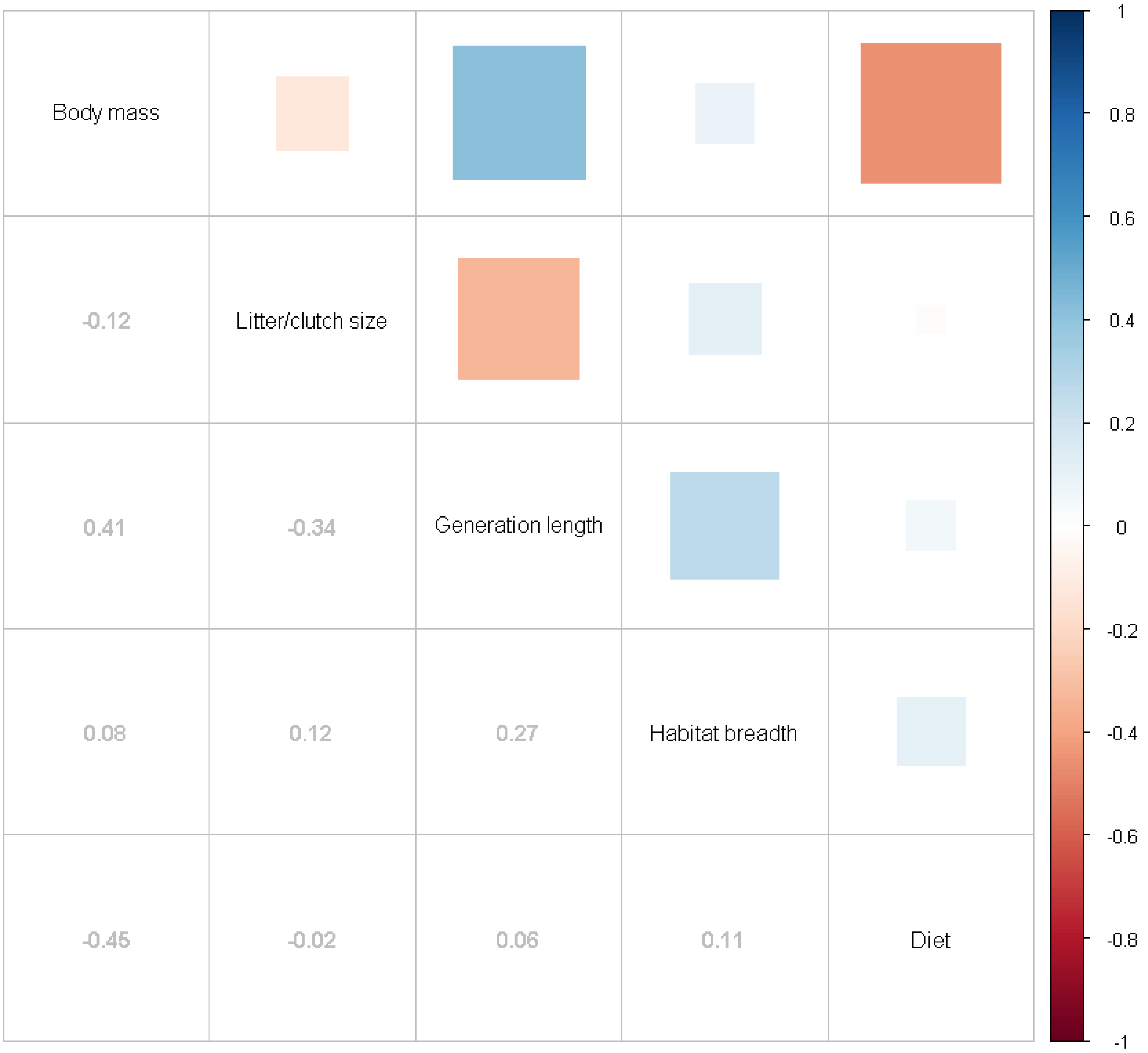

Supplementary Figure 6: Pairwise correlations between five traits for mammals and birds (15,484 species). Values are Pearson's correlation coefficients, with blue shades representing positive correlations and red shades reflecting negative correlations. Source data are provided as a Source Data file.

Supplementary Table 2: Percentage occupation by the observed ecological strategy space of the mean of 999 null strategy spaces generated from the assumptions of each null model, for each taxonomic group (all $P \leq 0.001$). We also tested across taxonomic groups, i.e., birds within mammal null strategy space. We found that birds are not completely nested within the null strategy space for mammals (and vice versa), due to extreme trait values that fall outside of the range of trait values for the other taxa (e.g., maximum generation length and litter/clutch size is greatest for birds, whereas maximum body mass and habitat breadth is greatest for mammals). Thus the different taxonomic groups do not completely share the same potential suite of trait combinations. In multi-dimensional space we find that 2.6% of the observed bird strategy space is unique compared to the null 1 mammal space, whereas 22.8% of the observed mammal space falls outside the null 1 bird space.

Null model*	Taxonomic group		
	Combined	Mammals	Birds
1: Traits uniformly distributed and independent from each other (approximately a hypercube)	9%	11%	11%
2: Traits normally distributed and independent from each other (approximately a hypersphere)	37%	43%	20%
3: Traits distributed as observed and independent from each other	62%	53%	71%
4: Traits normally distributed and correlated as observed (approximately a hyperellipsoid)	51%	74%	27%

* See[8] for full description and illustration of the null models

Supplementary Table 3: Contributions of different taxonomic and morphological groups to ecological strategy space. Volant mammals (bats [Chiroptera]; 1,103 species) may be expected to extend the mammalian ecological strategy space, due to strong ecomorphological differences to non-volant mammals. However we find that only 1.3% of the ecological strategy space occupied by mammals is attributable to bats. Thus, although bats may occupy a distant region of the ecological strategy surface compared to non-volant mammals, the ability of the hypervolume approach to model holes and disjunctions accounts for this separation. In addition, bats are densely packed in ecological strategy space, suggesting high convergence among strategies - reflecting previous spatial results, where bats showed high phylogenetic divergence but low ecological divergence[9]. In contrast, non-volant birds (predominantly ratites, penguins and flightless rails; 57 species) had a much lower density of 1.0 species SD^{-5}, implying high strategy divergence among flightless birds.

Group	No. of species	Volume (SD^5)	Unique volume	Density (species SD^{-5})
Mammals	5232	881	51% (unique component of all mammals and birds)	5.9
Birds	10252	534	19% (unique component of all mammals and birds)	19.2
Volant mammals (bats; Chiroptera)	1103	27	1.3% (unique component of all mammals)	40.4
Non-volant birds (predominantly ratites, penguins and flightless rails)	57	58	1.7% (unique component of all birds)	1.0

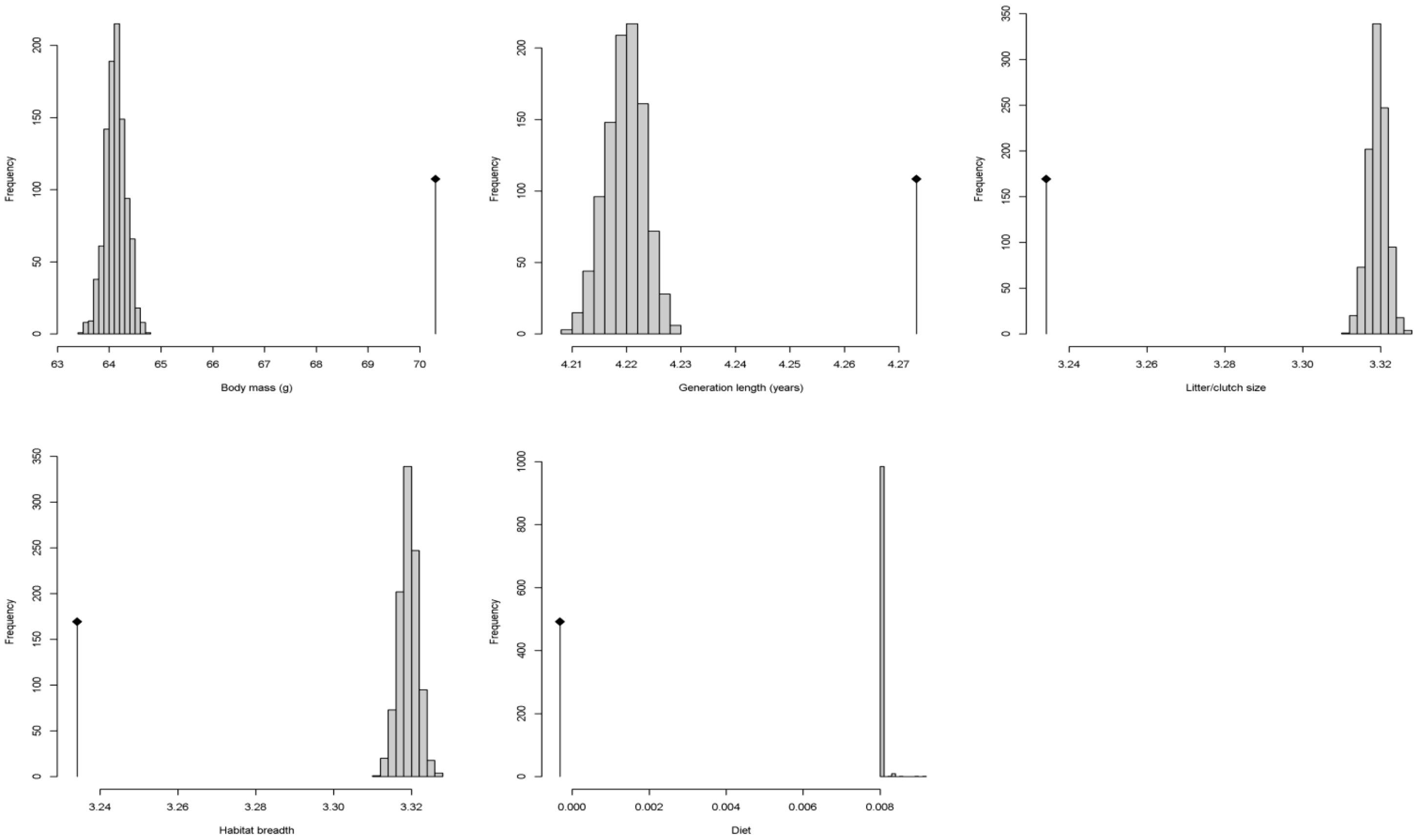

Supplementary Figure 7: Plots showing the results of the permutation tests, with histograms of the projected mean values (across 999 runs) and lollipops of the observed mean value per trait. When the lollipop is on the right of the histogram the trait is projected to decrease, whereas when the lollipop is on the left the trait is projected to increase. Source data are provided as a Source Data file.

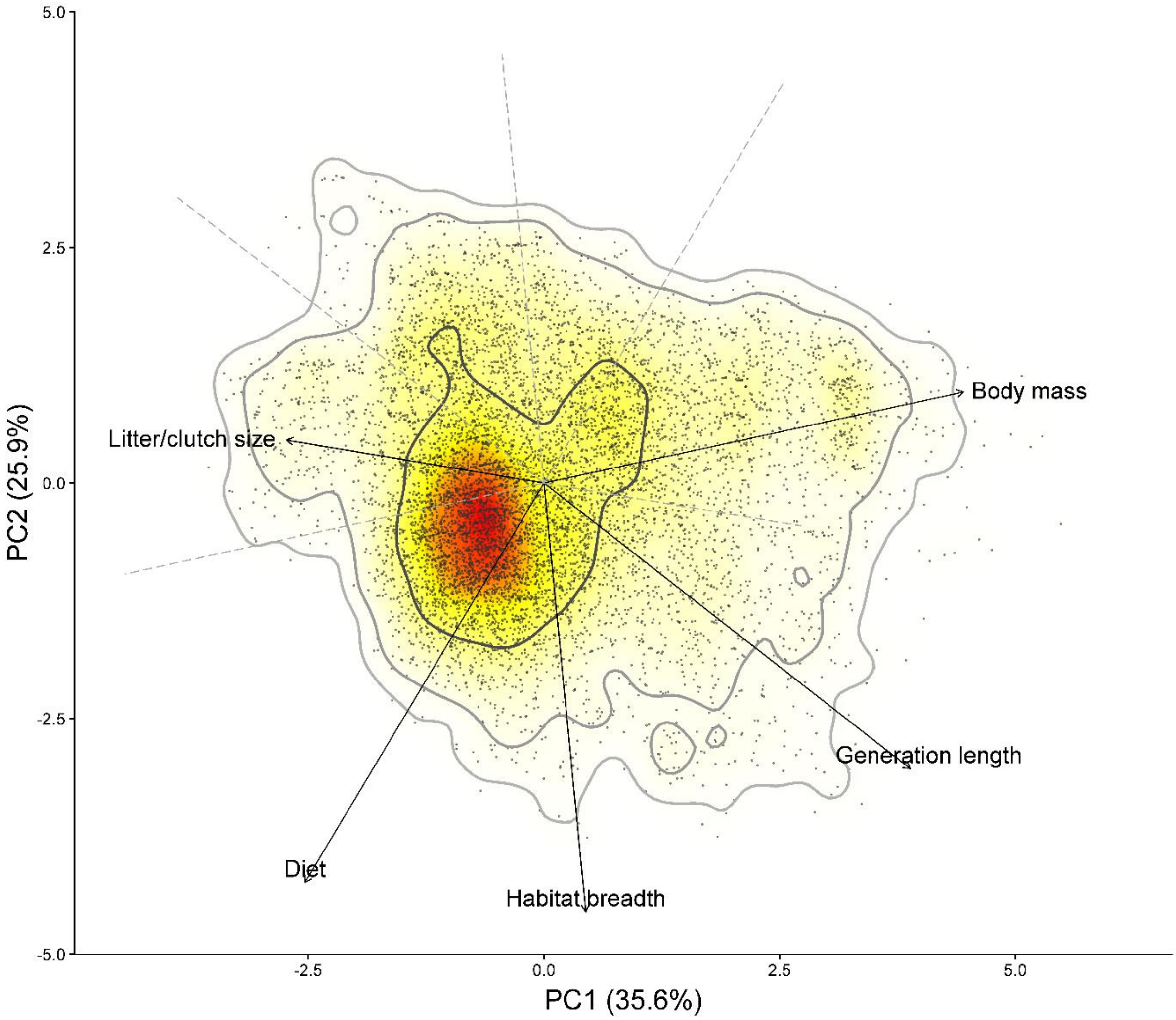

Supplementary Figure 8: The ecological strategy surface for mammals and birds (8,294 species) under the data deletion approach. Projection of extant terrestrial mammal and bird species with complete trait data (dots) on the surface defined by principal components (PC) 1 and 2. Source data are provided as a Source Data file.

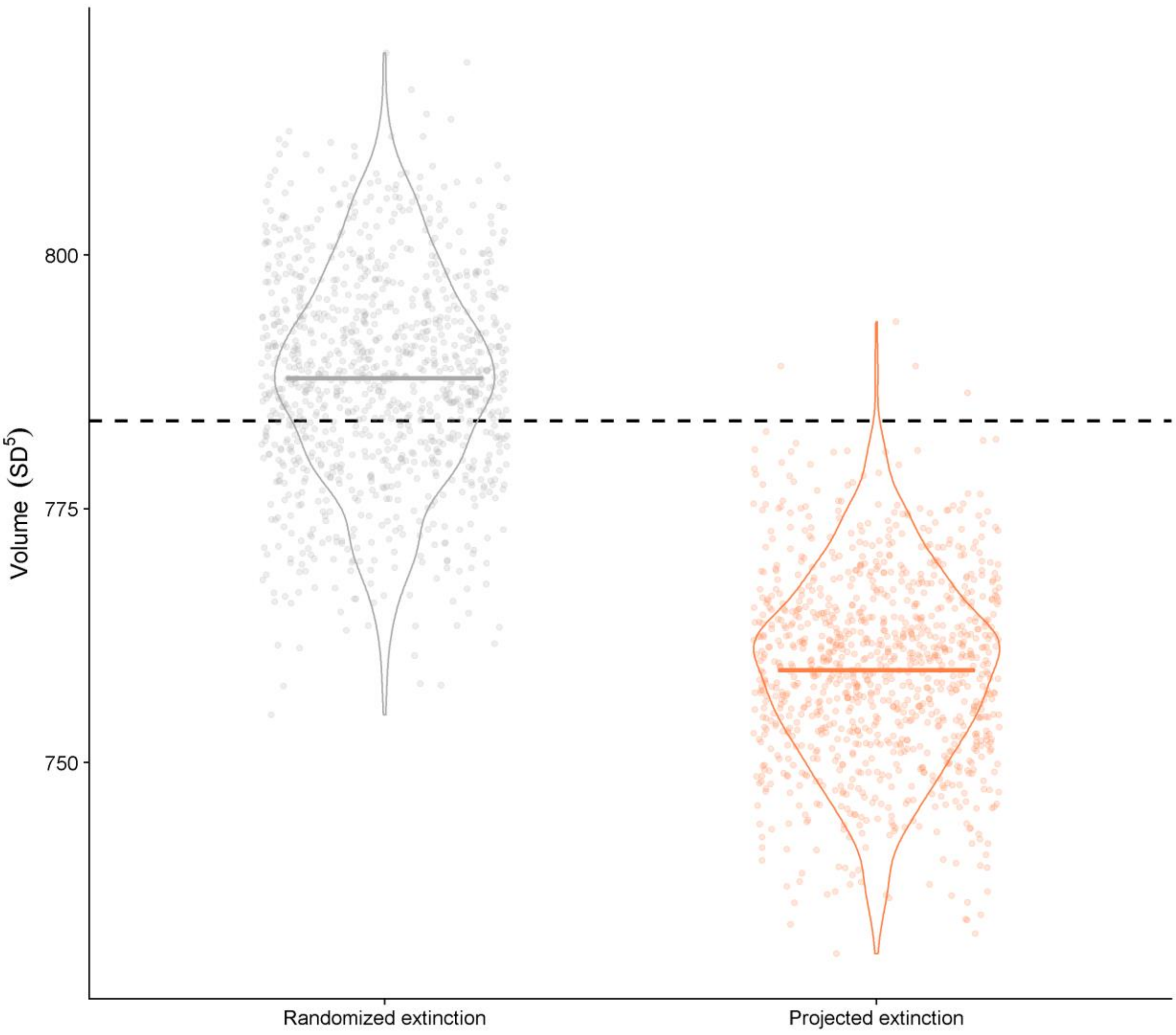

Supplementary Figure 9: The ecological strategy space for mammals and birds under 100-year extinction scenarios under the data deletion approach (8,294 species). The dashed horizontal line indicates the observed ecological strategy space (hypervolume), excluding missing-data species. 514 mammal and bird species are lost under both the projected and randomized extinction scenarios for the data deletion approach. Kolmogorov-Smirnov test: observed extinction mean under the data deletion approach = 784 SD^5, randomized extinction mean under the data deletion approach = 788 SD^5, projected extinction mean under the data deletion approach = 759 SD^5; $D = 0.85$, $P < 0.001$. Randomized compared to observed, effect size = +4.2 [95% CI: +23.9, -16.9] SD^5, projected compared to observed, effect size = -24.5 [-6.4, -43.8] SD^5. Source data are provided as a Source Data file.

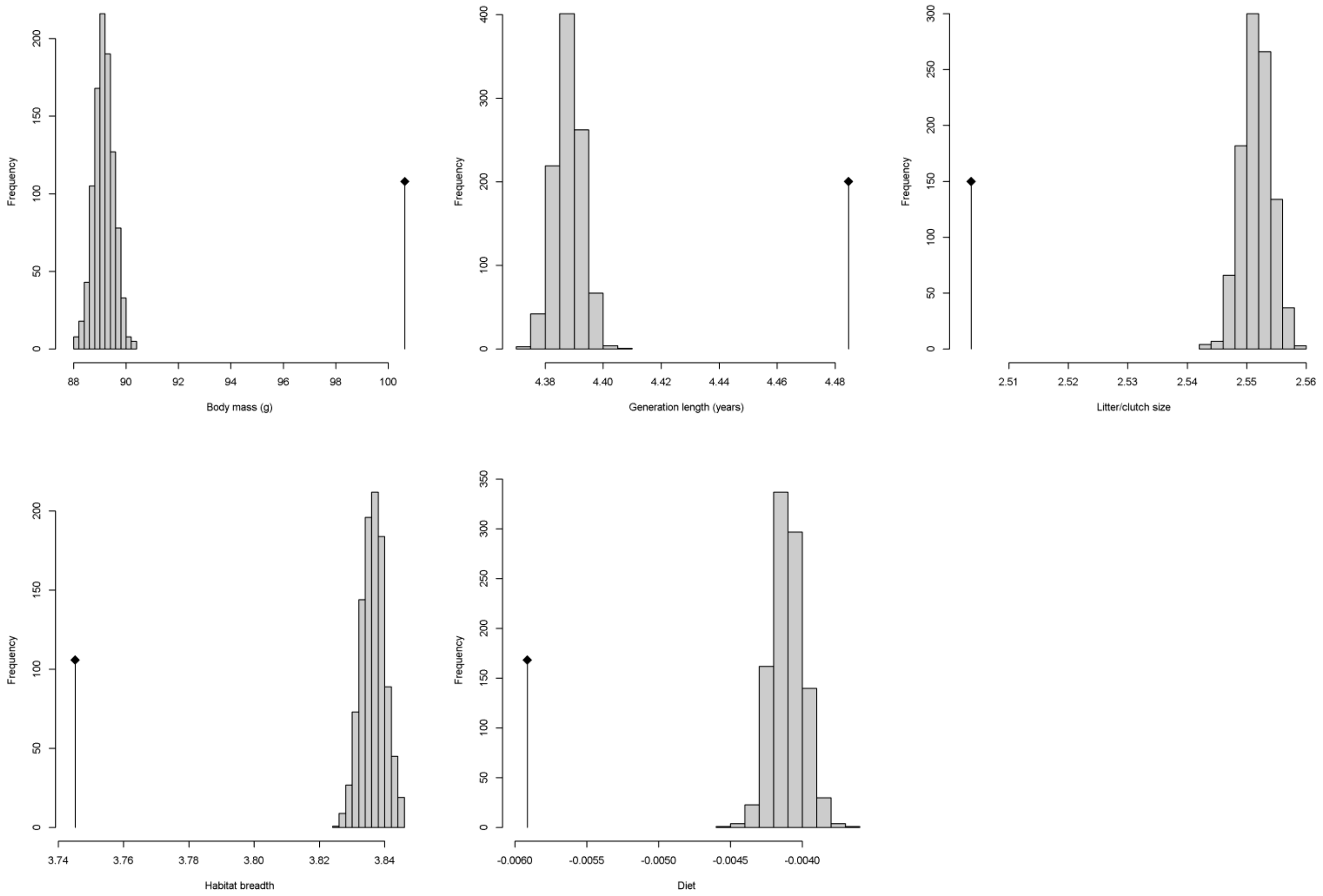

Supplementary Figure 10: Plots showing the results of the permutation tests under the data deletion approach (8,294 species), with histograms of the projected mean values (across 999 runs) and lollipops of the observed mean value per trait. Source data are provided as a Source Data file.

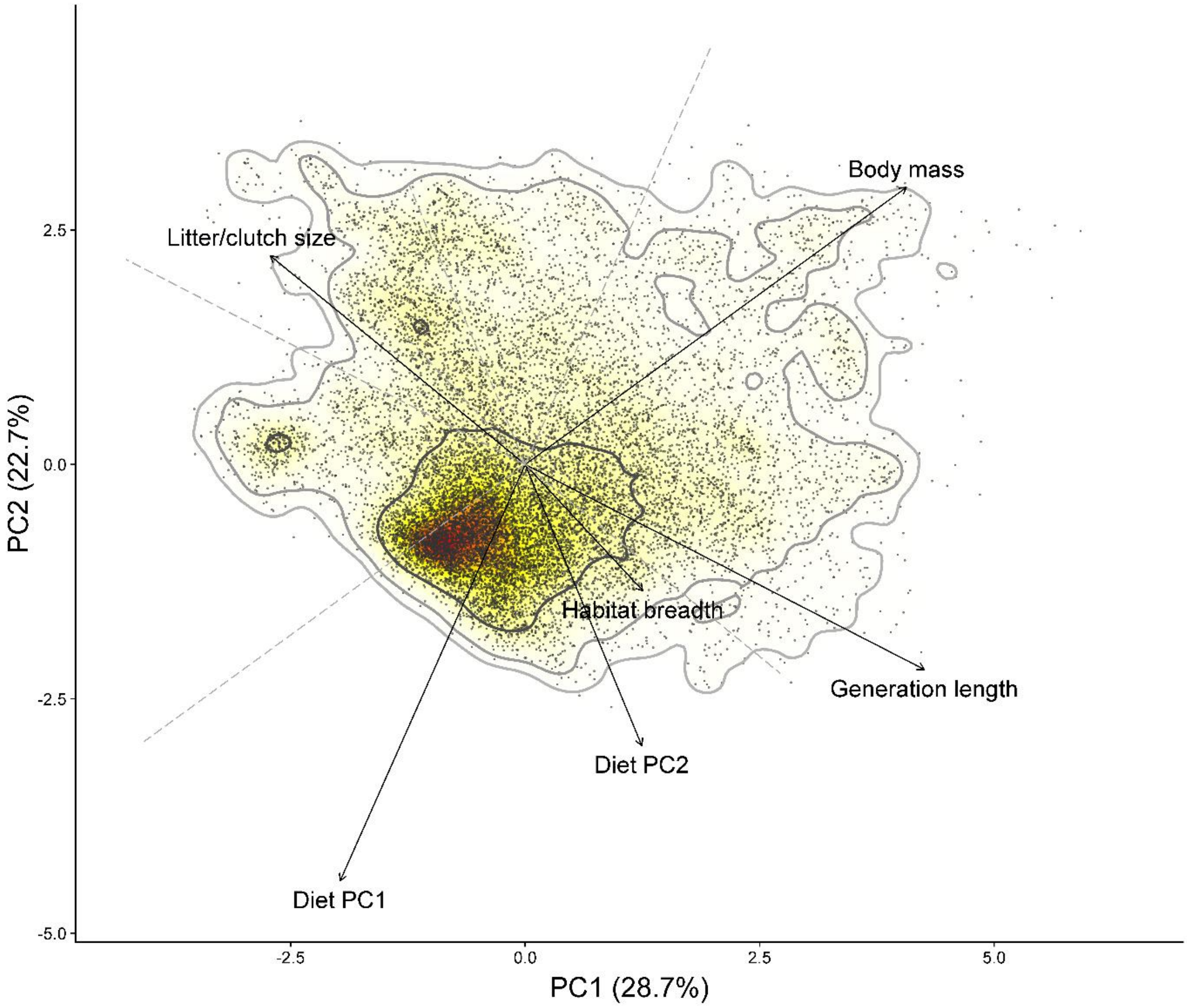

Supplementary Figure 11: The ecological strategy surface for mammals and birds (15,484 species) when including two synthetic diet traits (Supplementary Figure 4; Supplementary Methods). Source data are provided as a Source Data file.

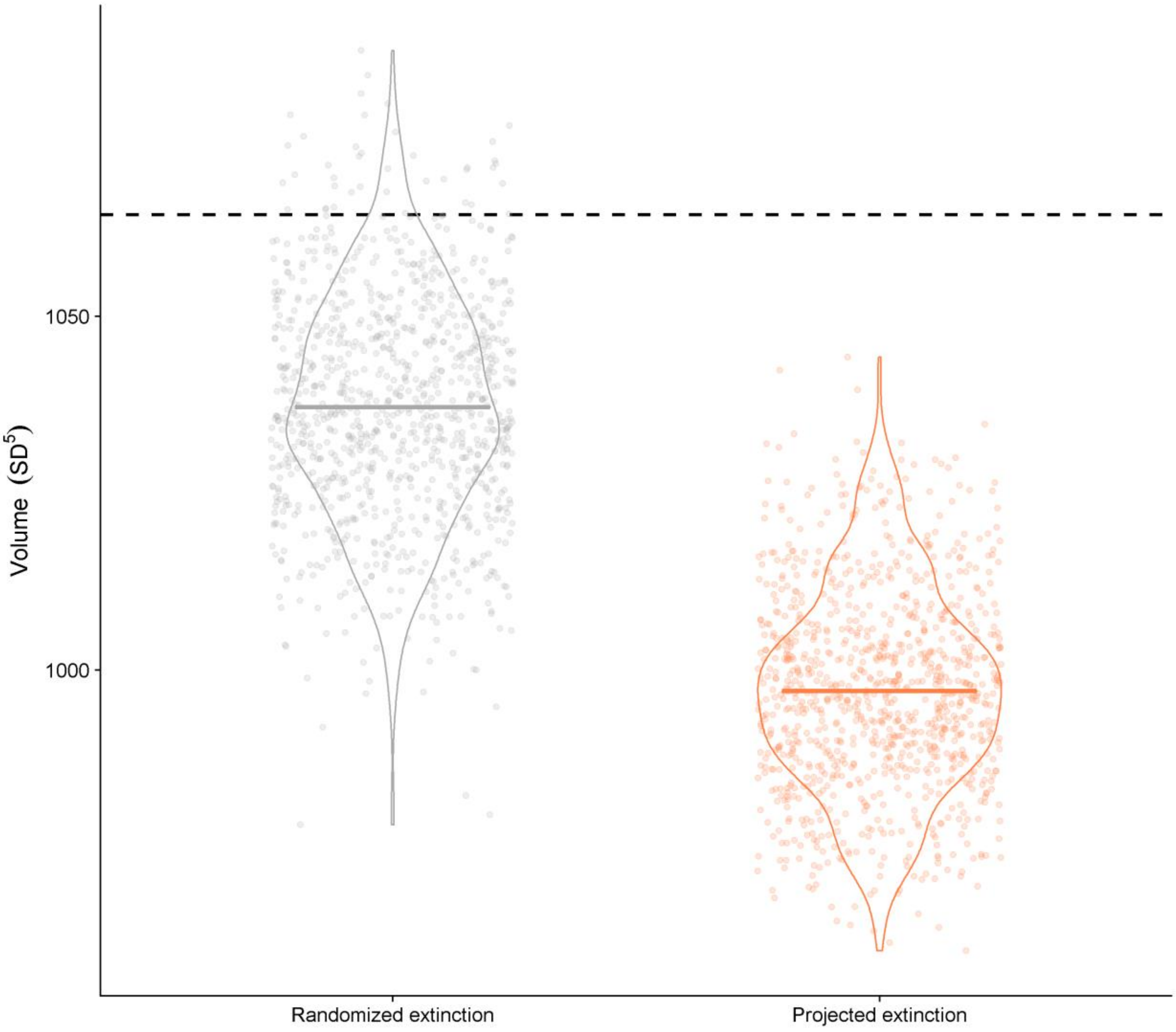

Supplementary Figure 12: The ecological strategy space for mammals and birds under 100-year extinction scenarios when excluding DD species (14,760 species). The dashed horizontal line indicates the observed ecological strategy space (hypervolume), excluding DD species. Kolmogorov-Smirnov test: observed extinction mean excluding DD species = 1064 SD^5, randomized extinction mean excluding DD species = 1037 SD^5, projected extinction mean excluding DD species = 997 SD^5; $D = 0.81$, $P < 0.001$. Randomized compared to observed, effect size = -27.2 [95% CI: +4.7, -56.9] SD^5, projected compared to observed, effect size = -67.3 [-38.1, -93.2] SD^5. Source data are provided as a Source Data file.

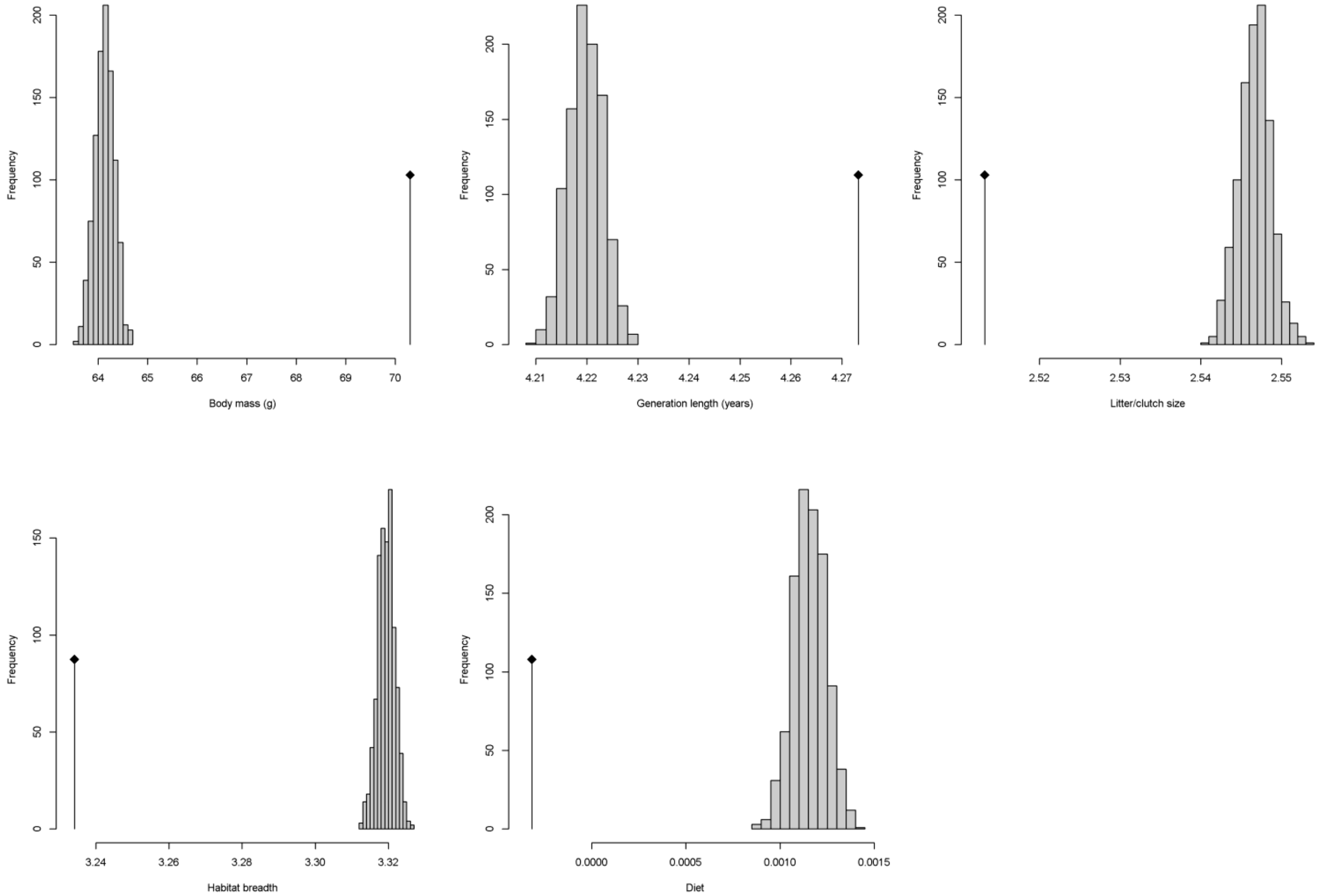

Supplementary Figure 13: Plots showing the results of the permutation tests when excluding DD species (14,760 species), with histograms of the projected mean values (across 999 runs) and lollipops of the observed mean value per trait. Source data are provided as a Source Data file.

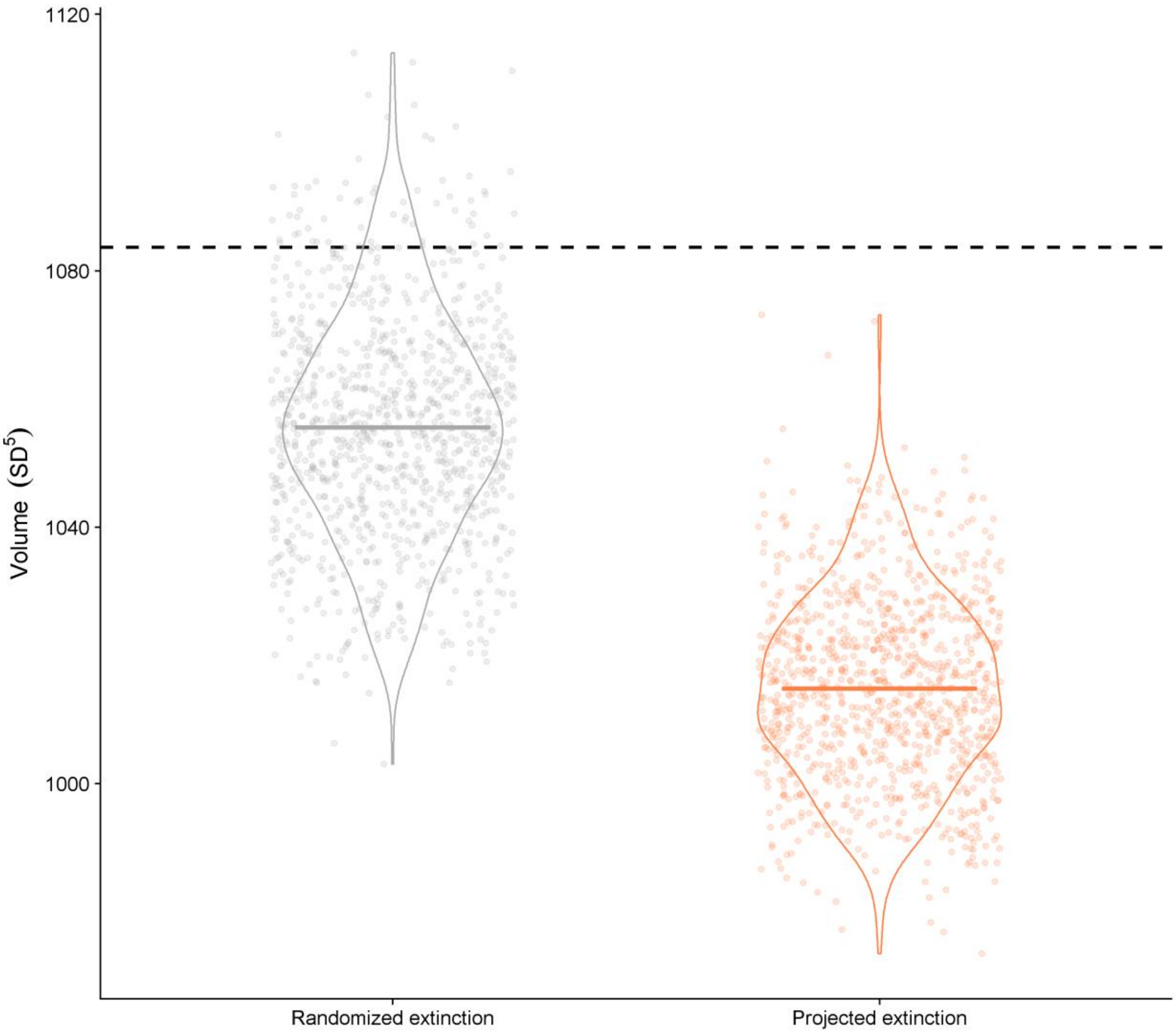

Supplementary Figure 14: The ecological strategy space for mammals and birds under 100-year extinction scenarios with predicted extinction probability for DD species (15,484 species). The dashed horizontal line indicates the observed ecological strategy space (hypervolume). 1,296 mammal and bird species are lost under both the projected and randomized extinction scenarios for the predicted DD species approach. Kolmogorov-Smirnov test: observed extinction mean for the predicted DD species approach = 1084 SD^5, randomized extinction mean for the predicted DD species approach = 1056 SD^5, projected extinction mean for the predicted DD species approach = 1015 SD^5; D = 0.80, P < 0.001. Randomized compared to observed, effect size = -28.1 [95% CI: +7.9, -61.6] SD^5, projected compared to observed, effect size = -68.9 [-39.5, -94.6] SD^5. Source data are provided as a Source Data file.

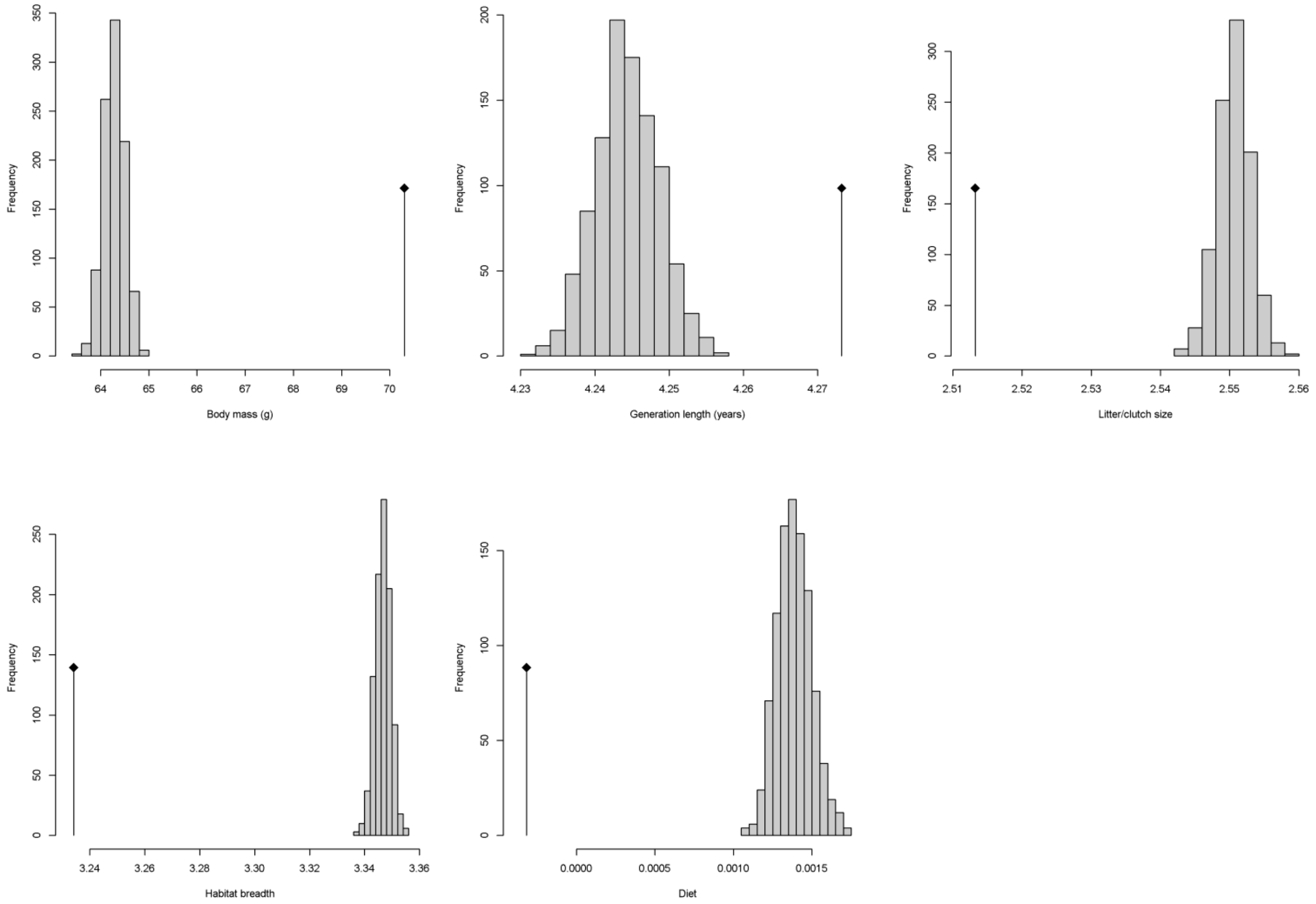

Supplementary Figure 15: Plots showing the results of the permutation tests when predicting extinction probability for DD species (15,484 species), with histograms of the projected mean values (across 999 runs) and lollipops of the observed mean value per trait. Source data are provided as a Source Data file.

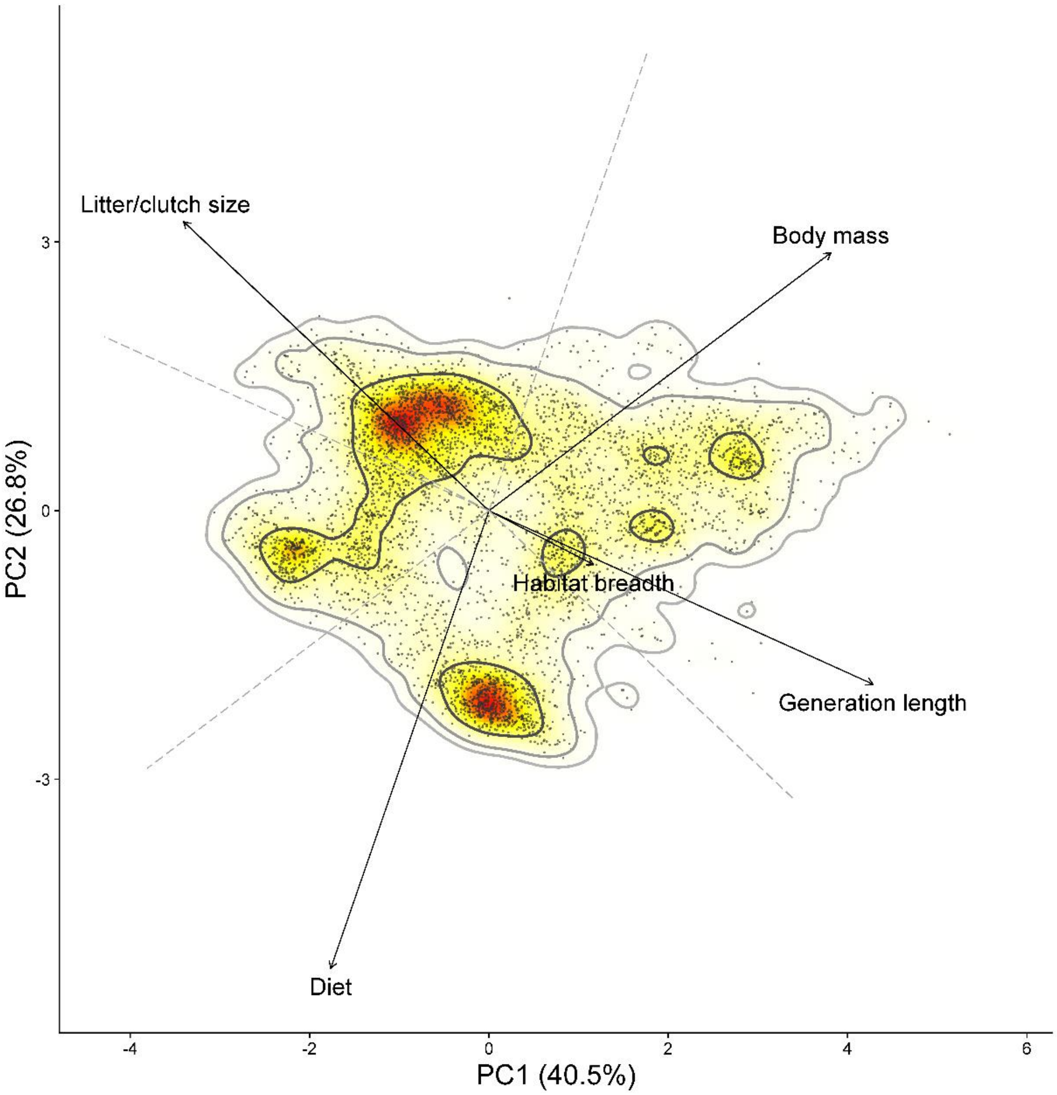

Supplementary Figure 16: Ecological strategy surface for mammals (5,232 species). Projection of extant terrestrial mammal species (grey dots) on the surface defined by principal components (PC) 1 and 2 (mean values across 25 imputed datasets; Supplementary Methods). Source data are provided as a Source Data file.

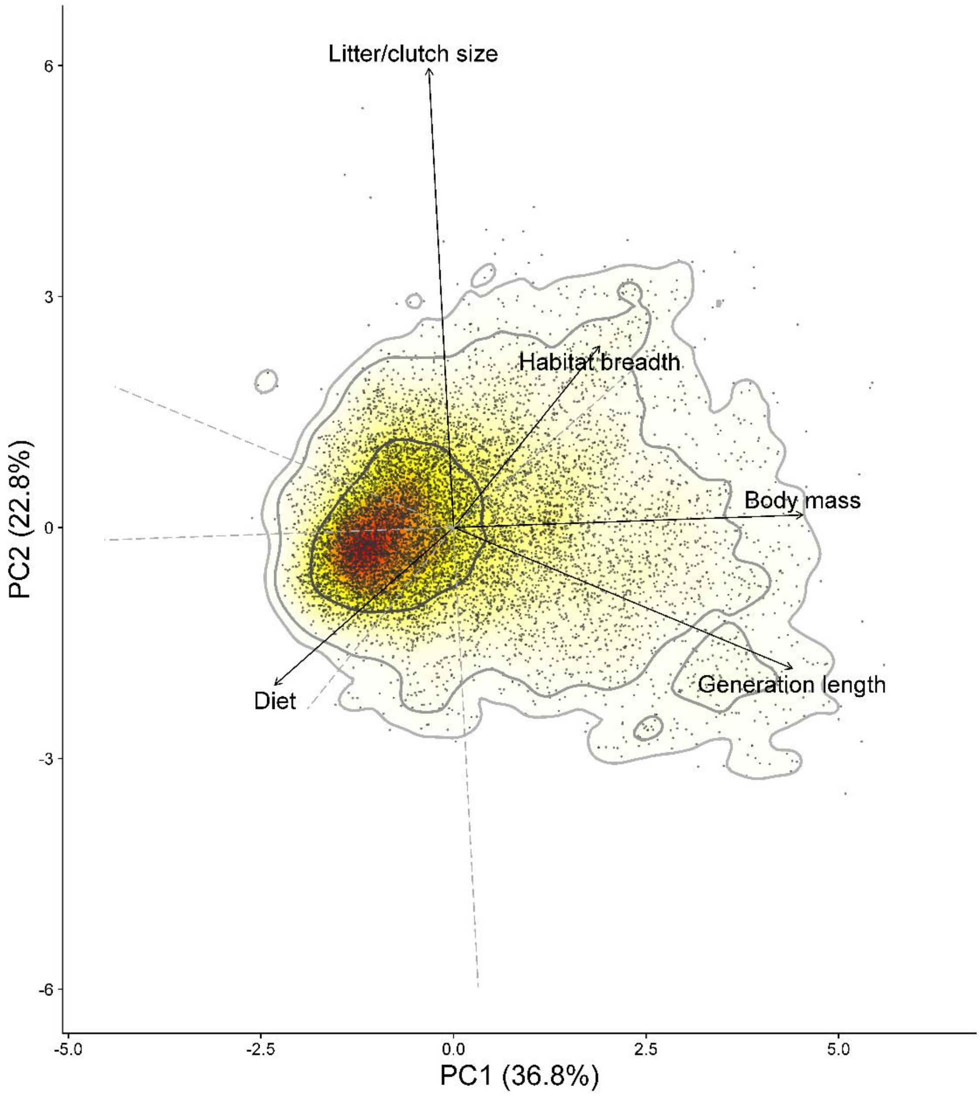

Supplementary Figure 17: Ecological strategy surface for birds (10,252 species). Projection of extant bird species (grey dots) on the surface defined by principal components (PC) 1 and 2 (mean values across 25 imputed datasets; Supplementary Methods). Source data are provided as a Source Data file.

Supplementary references

1. Cooke, R. S. C., Bates, A. E. & Eigenbrod, F. Global trade-offs of functional redundancy and functional dispersion for birds and mammals. *Glob. Ecol. Biogeogr.* **28,** 484–495 (2019).

2. Wilman, H. *et al.* EltonTraits 1.0: Species-level foraging attributes of the world's birds and mammals. *Ecology* **95,** 2027 (2014).

3. Purvis, A., Gittleman, J. L., Cowlishaw, G. & Mace, G. M. Predicting extinction risk in

declining species. *Proc. R. Soc. B Biol. Sci.* **267,** 1947–1952 (2000).

4. Veron, S. *et al.* Integrating data-deficient species in analyses of evolutionary history loss. *Ecol. Evol.* **6,** 8502–8514 (2016).

5. Bland, L. M., Collen, B., Orme, C. D. L. & Bielby, J. Predicting the conservation status of data-deficient species. *Conserv. Biol.* **29,** 250–259 (2015).

6. Isaac, N. J. B., Redding, D. W., Meredith, H. M. & Safi, K. Phylogenetically-informed priorities for amphibian conservation. *PLoS One* **7,** e43912 (2012).

7. Smith, F. A., Smith, R. E. E., Lyons, S. K. & Payne, J. L. Body size downgrading of mammals over the late Quaternary. *Science* **360,** 310–313 (2018).

8. Díaz, S. *et al.* The global spectrum of plant form and function. *Nature* **529,** 167–171 (2016).

9. Mazel, F. *et al.* Prioritizing phylogenetic diversity captures functional diversity unreliably. *Nat. Commun.* **9,** 2888 (2018).
