# Supplementary material for: Projected losses of global mammal and bird ecological strategies
Source: Nat Commun. 2019 May 23;10:2279. doi: 10.1038/s41467-019-10284-z (PMC6533255; doi:10.1038/s41467-019-10284-z)
Supplement: Supplementary file 3 — Reporting Summary [file 41467_2019_10284_MOESM3_ESM.pdf]

natureresearch

Corresponding author(s): Robert Cooke

Last updated by author(s): Apr 23, 2019

Reporting Summary

Nature Research wishes to improve the reproducibility of the work that we publish. This form provides structure for consistency and transparency in reporting. For further information on Nature Research policies, see Authors & Referees and the Editorial Policy Checklist .

Statistics

For all statistical analyses, confirm that the following items are present in the figure legend, table legend, main text, or Methods section.

n/a	Confirmed	
☐	☒	The exact sample size (*n*) for each experimental group/condition, given as a discrete number and unit of measurement
☐	☒	A statement on whether measurements were taken from distinct samples or whether the same sample was measured repeatedly
☐	☒	The statistical test(s) used AND whether they are one- or two-sided<br>*Only common tests should be described solely by name; describe more complex techniques in the Methods section.*
☒	☐	A description of all covariates tested
☐	☒	A description of any assumptions or corrections, such as tests of normality and adjustment for multiple comparisons
☐	☒	A full description of the statistical parameters including central tendency (e.g. means) or other basic estimates (e.g. regression coefficient) AND variation (e.g. standard deviation) or associated estimates of uncertainty (e.g. confidence intervals)
☐	☒	For null hypothesis testing, the test statistic (e.g. *F*, *t*, *r*) with confidence intervals, effect sizes, degrees of freedom and *P* value noted<br>*Give P values as exact values whenever suitable.*
☒	☐	For Bayesian analysis, information on the choice of priors and Markov chain Monte Carlo settings
☒	☐	For hierarchical and complex designs, identification of the appropriate level for tests and full reporting of outcomes
☐	☒	Estimates of effect sizes (e.g. Cohen's *d*, Pearson's *r*), indicating how they were calculated

Our web collection on statistics for biologists contains articles on many of the points above.

Software and code

Policy information about availability of computer code

Data collection	The dataset was previously compiled, and is available here: https://figshare.com/articles/Global_trade-offs_of_functional_redundancy_and_functional_dispersion_for_birds_and_mammals/5616424, with additional data provided here: http://doi.org/10.5061/dryad.gd0m3 and here: https://figshare.com/articles/Data_Paper_Data_Paper/3559887
Data analysis	All analyses were performed in R version 3.5.1. The simplified code, as an R notebook, is available on Github: https://github.com/03rcooke/hyper_pca

For manuscripts utilizing custom algorithms or software that are central to the research but not yet described in published literature, software must be made available to editors/reviewers. We strongly encourage code deposition in a community repository (e.g. GitHub). See the Nature Research guidelines for submitting code & software for further information.

Data

Policy information about availability of data

All manuscripts must include a data availability statement. This statement should provide the following information, where applicable:

- Accession codes, unique identifiers, or web links for publicly available datasets
- A list of figures that have associated raw data
- A description of any restrictions on data availability

The trait data were principally extracted from Cooke et al.28, which was compiled from four main databases14,63–65 and is available on figshare (https://figshare.com/articles/Global_trade-offs_of_functional_redundancy_and_functional_dispersion_for_birds_and_mammals/5616424; file: trait_data.csv). Generation length for mammals64 (http://doi.org/10.5061/dryad.gd0m3) and raw diet data14 (https://figshare.com/articles/Data_Paper_Data_Paper/3559887) were additionally compiled here. Generation length for birds was supplied by BirdLife but restrictions apply to these data, which were used under license for the current study. However, these data can be manually downloaded from the BirdLife website (http://datazone.birdlife.org/species/search). The code and data (without generation length due to data restrictions) to replicate our analyses is available on Github: https://github.com/03rcooke/hyper_pca.
In addition, the source data underlying Figs. 1, 2 and 3 and Supplementary Figures 1, 2, 3, 4, 5, 6, 7, 8, 9, 10, 11, 12, 13, 14, 15, 16 and 17 are provided as a Source

Data file.

Field-specific reporting

Please select the one below that is the best fit for your research. If you are not sure, read the appropriate sections before making your selection.

☐ Life sciences ☐ Behavioural & social sciences ☒ Ecological, evolutionary & environmental sciences

For a reference copy of the document with all sections, see nature.com/documents/nr-reporting-summary-flat.pdf

Ecological, evolutionary & environmental sciences study design

All studies must disclose on these points even when the disclosure is negative.

Study description	For this study we ordinated (Principal Components Analysis) all extant terrestrial mammals and birds based on their traits to summarize their ecological strategies. We also constructed 5-dimensional ecological strategy spaces, via hypervolume estimation, to reveal the overlap and convergence between mammals and birds; and finally we modelled the impact of future projected extinctions on the global ecological strategy space of mammals and birds.
Research sample	We analyzed all 15,484 extant terrestrial species of mammals and birds.
Sampling strategy	The sample size was designed to be globally inclusive, i.e., all living land mammals and birds were included in the study
Data collection	The majority of the data was previously compiled here: https://figshare.com/articles/Global_trade-offs_of_functional_redundancy_and_functional_dispersion_for_birds_and_mammals/5616424, with additional data on generation length and diet compiled here from referenced sources and available online.
Timing and spatial scale	Trait data was compiled in 2016 for all species globally
Data exclusions	Trait data were not available for all species. The common practice of using only species with complete data (data-deletion approach) not only reduces sample size and consequently the statistical power of any analysis, but may also introduce bias. Instead, to achieve complete species-trait coverage we imputed missing trait data. The data deletion approach was performed for comparative purposes (8,294 species).
Reproducibility	All data and code to run the analyses has been made available or links to where it is freely available are supplied, so that the analyses could be reproduced: https://github.com/03rcooke/hyper_pca. Due to the stochastic nature of the hypervolume algorithm the results would not be identical if repeated, but the use of 999 replicates should mean that results are qualitatively equivalent.
Randomization	We used Monte Carlo permutations to randomize the trait data under the assumptions of four alternative null models, with 999 replicates. We also replicated both the projected and randomized (random with respect to species identity/traits) extinction scenarios 999 times.
Blinding	All analytical decisions, code and procedures were implemented and justified (see Supplementary Methods) before the results were revealed in their entirety.

Did the study involve field work? ☐ Yes ☒ No

Reporting for specific materials, systems and methods

We require information from authors about some types of materials, experimental systems and methods used in many studies. Here, indicate whether each material, system or method listed is relevant to your study. If you are not sure if a list item applies to your research, read the appropriate section before selecting a response.

Materials & experimental systems

n/a	Involved in the study
☒	☐ Antibodies
☒	☐ Eukaryotic cell lines
☒	☐ Palaeontology
☒	☐ Animals and other organisms
☒	☐ Human research participants
☒	☐ Clinical data

Methods

n/a	Involved in the study
☒	☐ ChIP-seq
☒	☐ Flow cytometry
☒	☐ MRI-based neuroimaging
